# Modeling the Tensile Behavior of Fiber-Reinforced Strain-Hardening Cement-Based Composites: A Review

**DOI:** 10.3390/ma16093365

**Published:** 2023-04-25

**Authors:** Paula de Oliveira Ribeiro, Pablo Augusto Krahl, Ricardo Carrazedo, Luís Filipe Almeida Bernardo

**Affiliations:** 1 Department of Structural Engineering, Federal University of Juiz de Fora, Rua José Lourenço Kelmer, s/n São Pedro, Juiz de Fora 36036-900, MG, Brazil; paula.oliveira@ufjf.br; 2 Department of Civil Engineering, Mackenzie Presbyterian University, Av. Brasil, 1220-Jardim Guanabara, Campinas 13073-148, SP, Brazil; pablo.krahl@mackenzie.br; 3 School of Engineering of São Carlos, University of Sao Paulo, Av. Trabalhador Saocarlense, 400, Sao Carlos 13566-590, SP, Brazil; carrazedo@usp.br; 4 Department of Civil Engineering and Architecture, University of Beira Interior, 6201-001 Covilhã, Portugal

**Keywords:** Strain-Hardening Cement-Based Composites (SHCCs), tensile behavior, modeling methodologies

## Abstract

Strain-Hardening Cement-Based Composites (SHCCs) exhibit high toughness and durability, allowing the design of resilient structures. Despite the exceptional properties of SHCC and the current modeling techniques, the widespread use of the composite is limited. One limiting factor is developing and validating analytical models that could be used for optimizing mixes and designing structural elements. Furthermore, the composite mechanical response is complex and depends on several phenomena, such as fiber pullout, fiber orientation and distribution, size effect, fiber content, group effect, embedding length, fiber dimensions, and matrix strength. In this context, this research presents the state-of-the-art on the micro- and mesomechanisms occurring in SHCC during cracking and robust techniques to predict its tensile behavior accounting for such phenomena already proved experimentally. The study is relevant for designers and the scientific community because it presents the gaps for the research groups to develop new investigations for consolidating SHCC, which is a material to produce resilient structures.

## 1. Introduction 

Strain-Hardening Cement-Based Composites (SHCCs) exhibit high ductility, toughness, and durability properties. The quasiductile behavior can be achieved by adding well-distributed fibers, bridging the multiple fine cracks (Jun and Mechtcherine, 2010 [1]). Furthermore, optimizing the composite composition provides a dense mesostructure to the material. Therefore, very high strength can be reached (Fehling et al., 2014 [2]; Krahl, Carrazedo and El Debs, 2018 [3]; Duque and Graybeal, 2017 [4]). In addition, steady-state cracking propagation can also be obtained through design with a high deformation capacity.

The mechanical response of SHCC is complex. First, cracking and location are typically controlled by microscopic defects in the matrix (Curosu et al., 2017 [5]; Jun and Mechtcherine, 2010 [1]). After that, several mechanisms between the fibers and the matrix determine the material behavior in the postpeak, such as the interaction between the fibers (group effect), fiber pullout (fiber–matrix interface), spalling and snubbing effect, fiber orientation, and distribution. Stress is transferred from the fiber to the matrix at cracks, and if steady state criteria are attended, multiple cracking can occur. In addition, production and casting are other essential factors that interfere with fiber distribution and, consequently, govern the material’s mechanical behavior (Duque and Graybeal, 2017 [4]). Such influence boosted research to evaluate the relationship between fiber distribution and orientation for the better use of material strength and understand statistically how much of the strength can be effectively used in the design. Analysis of fiber distribution with techniques such as image analysis of cut sections, X-ray, and CT (computed tomography) scan provide the fiber orientation properties associated with the casting process and material tensile behavior. These studies show that depending on the fiber content and orientation, the material can present a strain-hardening or strain-softening behavior.

**Figure 1:**
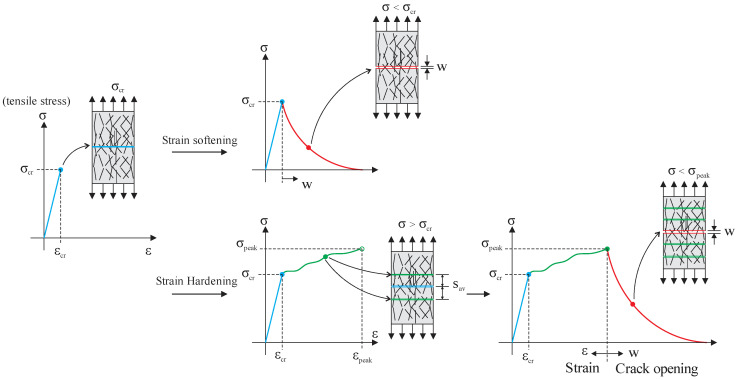
SHCC tensile behavior.

The strain-hardening regime is characterized by multiple cracks and a stress redistribution that increases strength and ductility before strain localization at one crack. The strain-hardening response can be divided into three parts. In part 1, the behavior is elastic, followed by the development of microcracking and the beginning of fiber pullout, as they act mainly in the postcracking phase by forming bridges between the crack faces. In part 2, there is strain-hardening behavior with multiple cracks. Finally, in part 3, strain localization results in softening behavior (Wille and Naaman, 2010 [6]) (see Figure 1). In the strain-softening response, part 2 does not occur. The meaning of the parameters in Figure 1 are as followings: *σ_cr_* is the cracking stress, *ε_cr_* is the strain corresponding to *σ_cr_*, *w* is the main crack width, *σ_peak_* is the maximum stress, and *ε_peak_* is the corresponding strain.

The addition of fibers to SHCC delays crack coalescence and increases toughness (Krahl, Carrazedo and Debs, 2018 [3]). Brittle and quasibrittle solids have low toughness capacity in tension, and adding fibers promotes an extrinsic fracture mechanism, reducing the stress intensity in the crack tips. Hence, fiber reinforcement is an efficient way to convert brittle concrete into a pseudoductile material (Li, Wang and Backer, 1990 [7]) by designing the material with fracture mechanics criterion (Li, 2019 [8]) or with critical fiber volume fraction. There are fibers with several types of materials (carbon, steel, polymers, glass) and shapes for developing cement-based materials with residual capacity. For example, shapes available are hooked fibers (Abdallah, Fan, and Zhou, 2016 [9]; Cao and Yu, 2018 [10]; Gebuhr et al., 2019 [11]), arched (Yoo, Chun, and Kim, 2020 [12]), corrugated (Wu, Khayat, and Shi, 2018 [13]; Zhang, Ji, Lin, 2019 [14]), twisted (Wille and Naaman, 2010 [15]), and flattened-end fibers (Abu-Lebdeh et al., 2011 [16]). However, strain-hardening composites are developed based on interfacial fiber–matrix optimization. One of the goals is to reduce mechanical anchorage or excessive bond for fiber slip instead break, which gives more deformation capacity—composites such as ECC and UHPC typically use straight synthetic and steel fibers, respectively. 

Despite the positive effect of adding fibers to SHCC, its mechanical behavior depends significantly on the fiber distribution and orientation associated with the production process. The material rheology strongly influences the fiber orientation, the casting procedure adopted, and the formwork geometry; for example, the wall effect orients fibers in specimens with small cross-sections (Abrishambaf, Pimental and Nunes, 2019 [17]), and circular panel fibers tend to align perpendicularly to the radial flow in circular panels when concrete is poured from the center (Zhou and Uchida, 2017 [18]).

In practice, the preferential fiber orientation along a specific direction probably occurs, leading to the anisotropic behavior of the concrete (Oliveira, 2019 [19]), making the material directionally dependent. Such a trend needs to be understood to predict the structural behavior better. Furthermore, models must consider fiber orientation and distribution and evaluate the cast process and shape of the structural elements that differ from laboratory samples (Abrishambaf, Pimentel and Nunes, 2019 [17]).

There are several modeling techniques to consider fiber orientation, including multiphase modeling (Bitencourt Jr et al., 2019 [20]; Qsymah, 2016 [21]; Cunha, Barros and Sena-Cruz, 2011 [22]; Soetens et al., 2012 [23]), inverse analysis of experimental results (NF P18-470, 2016 [24]), homogenization theory (Dutra, Maghous and Campos Filho, 2013 [25]; Qsymah, 2016 [21]), and analytical formulations based on micromechanical phenomena (Abrishambaf, Pimentel and Nunes, 2019 [17]; Lee, Cho and Vecchio, 2011 [26]; Li, 1992 [27]). The French standard (NF P18-470, 2016 [24]) recommends designing Ultrahigh-Performance Fiber-Reinforced Concrete (UHPFRC) structures considering fiber orientation. The standard introduces an orientation factor *K*, which expresses the effect of the placement of UHPFRC in the structure, which can be determined from a bending test. From the published literature, it is consensual that fiber orientation must be a design parameter, which is highlighted in the proposed review.

Despite the exceptional properties of SHCC and the current modeling techniques, the widespread use of the composite is limited. One limiting factor is developing specific and validated analytical models that could be used for designing. In addition, many researchers still use isotropic models implemented in nonspecialized finite element commercial software to simulate this material. Therefore, it is necessary to develop models that consider fiber-induced anisotropy and micromechanical phenomena in evaluating the performance of structural elements of SHCC (Duque and Graybeal, 2017 [4]).

In this paper, a review of the main reported micromechanical phenomena and current modeling methodologies is performed. Many studies presented in this paper refer to UHPFRC, a particular class of SHCC. The toughening mechanisms that influence the material behavior of the composite are also discussed. In addition, fiber pullout, orientation and distribution, size effect, fiber content, group effect, embedding length, fiber dimensions, and matrix strength are presented, as are the modeling approaches to modeling fiber-reinforced composites.

## 2. Factors Influencing the Mechanical Behavior of the Material

### 2.1. Fiber Pullout

**Figure 2:**
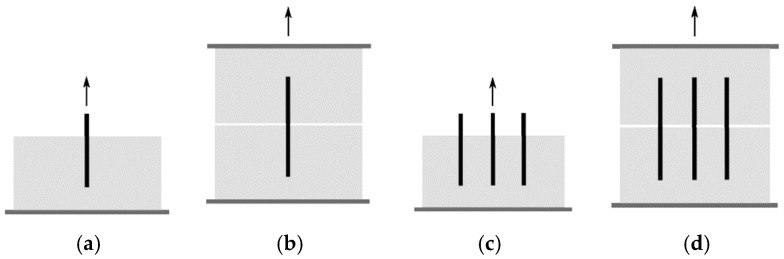
(**a**) Single-sided specimen and single fiber; (**b**) double-sided specimens and single fiber; (**c**) single-sided specimen and multiple fibers; (**d**) double-sided specimens and multiples fibers.

The mechanical properties of Fiber-Reinforced Cement-Based Composites (FRCC) significantly depend on the stress transfer efficiency during fiber bridging. The fiber pullout test allows for assessing the interfacial properties between fiber and cement-based matrixes, which helps to predict and optimize the mechanical behavior of the composite. The fiber–matrix interface properties are affected by the cement hydration (curing time and age), matrix density (packing), and surface treatment of the fiber, coating or mechanical crimping (Du et al., 2021 [28], Zhou and Qiao, 2019 [29]; Li, Wang and Backer, 1990 [7]). The test is usually performed with the fiber embedded in both sides of the sample or on one side. Furthermore, it can be made from a single fiber or multiple fibers, as illustrated in Figure 2. Tests with multiple fibers depict an interaction between the fibers depending on the distance interfering with the load they supported. Fu et al. (2000) [30] emphasize that research on multiple fiber pullout is critical for understanding stress transfer in composites because the stress field resulting from shear transfer between each fiber and matrix during pullout interacts with the fields of the neighbor fibers, typically reducing the pullout load. Kim and Yoo (2019) [31] investigated the multiple fiber pullout experimentally. The authors examined the interaction between neighboring fibers embedded in Ultrahigh-Performance Concrete (UHPC). Straight, hooked, and twisted fibers were investigated. Four spaces between fibers were used to represent composites with volumetric fractions of 1%, 2%, and 7% of fibers, and the case of fiber bundle (clustering). The twisted steel fiber exhibited the highest pullout strength in the single fiber test, followed by hooked and straight steel fibers. There was a 22–30% decrease in the average bond strength in specimens tested with multiple fibers to the single fiber test. In the case of fiber without spacing (bundled), the test presented a decrease of 52% in load, that is, bundled fibers showed a much more significant reduction in bond strength than multiple fibers. Therefore, the bond strength is maximum for single fibers and minimum for bundled fibers. This trend occurs due to the difficulty of the matrix adhering to the fiber surface when fibers touch each other. Unfortunately, very little research has been explored in this regard.

The pullout test provides parameters used in the constitutive models for UHPFRC. The average bond strength (*τ*) is an essential fiber interface property and can be determined with Equation (1). The bond *τ* is determined from the maximum pullout load (*P*_max_), the embedded length at the test beginning (*l_e_*), and fiber diameter (*d_f_*). The average bond strength *τ* is an input parameter to obtain the pullout response with analytical models. The consideration of embedding length uniformly distributed in the 0 to *l_f_*/2 domain is usually assumed; thus, the average value of the embedding length is *l_f_*/4 (Abrishambaf, Pimentel and Nunes, 2017 [32]).

(1) $$\tau =\frac{P_{\max}}{\pi d_{f}l_{e}}$$

Li (2019) [8] developed an analytical expression for single fibers being pulled normally to the crack face *P*(*w*,*l_e_*), Equation (2a). The peak load corresponds to a slip of *u* = *u*_0_. After the maximum load, the pullout is represented by Equation (2b), which is the transition between bond and friction. The interfacial parameter *β* can be achieved by fitting the experimental fiber pullout curve. Furthermore, research suggests that the *β* parameter is influenced by the fiber inclination and fiber content in the matrix (Lei et al., 2021 [33]; Ribeiro, Krahl and Carrazedo, 2022 [34]).

(2a) $$P(u)=\pi \sqrt{\frac{E_{f}d_{f}^{3}(1+\eta )\tau u}{2}}\;\mathrm{for}\;u\leq u_{0}$$

(2b) $$P(u)=\pi d_{f}\tau (l_{e}-(u-u_{0}))\left(1+\beta \frac{u-u_{0}}{d_{f}}\right)\;\mathrm{for}\;u>u_{0}$$

where $u_{0}=\frac{2\tau l_{e}^{2}}{E_{f}d_{f}(1+\eta )}$.

The fiber pullout test must be performed with straight and inclined fibers, as most fibers have inclinations with the crack surface in the composite. Krahl et al. (2021) [35] tested fibers with inclinations of 0°, 30°, and 45° to the loading direction. The snubbing and spalling effect mechanisms occur in inclined fibers due to frictional stresses increasing at the exit point of the fiber cavity. The snubbing effect positively influences the pullout load. In contrast, the spalling effect is harmful because it reduces the fiber contact area and stress transfer capacity. Considering these two simultaneous effects with opposite influences on the test response, an optimal fiber orientation improves the material’s ultimate carrying capacity and ductility regarding energy absorption. The maximum pullout load of a steel fiber increases with the inclination until an angle of around 30°. Above this angle, a reduction in the maximum load occurs (Duque and Graybeal, 2017 [4]). Spalling prevails for more significant inclinations, and fibers can fail due to substantial shear forces.

**Figure 3:**
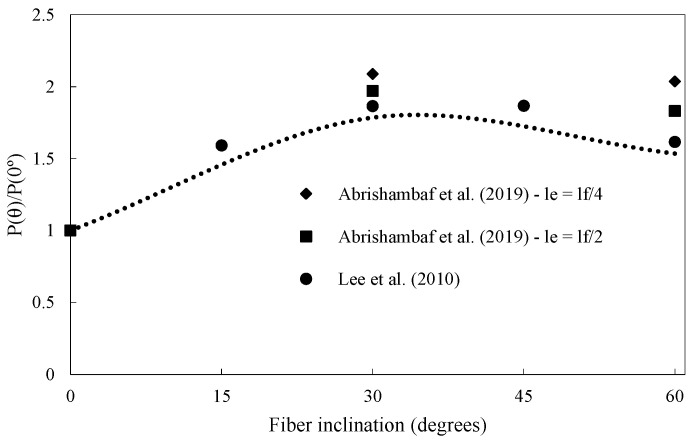
Influence of fiber inclination on the pullout.

The function *g*(*θ*) is used to correlate the inclined fiber pullout *P*(*θ*) with the straight fiber pullout *P*(*w*,*l_e_*), according to Equation (3). Li (1992) [27] adopted a function $g(\theta )=e^{f\theta }$, where *θ* is the fiber angle to crack face and *f* is the coefficient that accounts for the snubbing effect for nylon and polypropylene (PP) fibers. This coefficient can be thought of as an increase in the bond strength. In the case of steel fibers, the fiber inclination causes matrix spalling for large angles, which must be considered. Zhou and Qiao (2019) [29] and Lee, Kang, and Kim (2010) [36] used the equation $g(\theta )=e^{f\theta }\cos\theta^{k}$ for UHPFRC. The coefficient *f* represents the snubbing phenomena, and *k* the matrix spalling arising from the inclination of the steel fiber in the matrix. For steel fibers embedded in a plain UHPC matrix, Lee, Kang, and Kim (2010) [36] obtained the relationship between the bond strength and pullout angle resulting in *f* of 1.6 and *k* of 1.8. Figure 3 presents some results from the literature and the function *g*(*θ*) suggested by Lee, Kang, and Kim (2010) [36].

(3) $$P(\theta )=P(w,l_{e})g(\theta )$$

### 2.2. Matrix Strength

**Figure 4:**
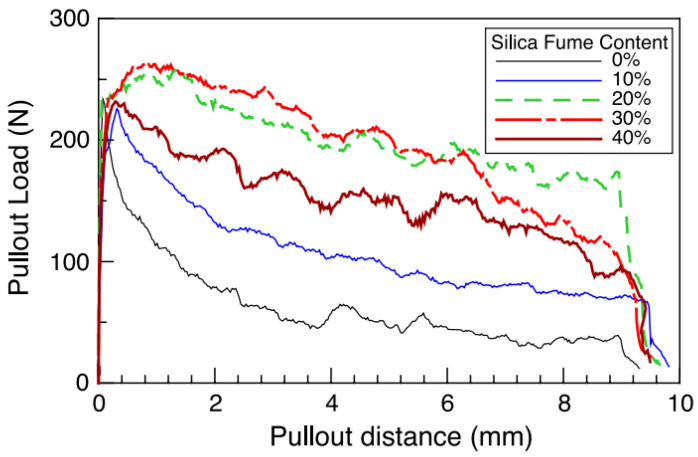
Fiber pullout curves for different fiber contents (Chan and Chu (2004) [37]).

Increasing matrix strength tends to refine and enhance the interface between fiber and matrix. For example, silica fume, which typically has an average dimension smaller than that of cement, densifies the interfacial transition zone between the two phases, increasing pullout load and the energy dissipated through fiber slippage. Chan and Chu (2004) [37] investigated the effect of incorporating silica fume in reactive powder concrete. They found that silica fume can improve fiber–matrix interfacial properties. Figure 4 (Chan and Chu (2004) [37]) shows the pullout response for different silica fume contents. The optimal silica fume was between 20% and 30%, as the content of 30% had no significant increase in bond compared to 20% addition, and higher values than 30 % had a deleterious effect. The limited positive effect is attributed to silica fume being the smallest particle, with high attracting forces tending to flocculate. Such a trend requires higher mixture energy than the compositions with smaller contents, reducing workability and diminishing the effectiveness of the particles packing the interface with the fiber surface. Therefore, the pozzolanic activities are reduced, and the interfacial properties are inferior to lower silica fume contents. 

Jewell et al. (2022) [38] performed pullout tests to investigate the bond between fibers and calcium sulfoaluminate (CSA) cement matrix. Fibers with differing Young’s Modulus and strengths were selected to test the fiber–matrix bond, such as polyvinyl-alcohol (PVA), PP, coated steel, and plain steel. Three matrices were selected: two sulfate-based cements, a commercial CSA cement and a CSA cement produced from coal-combustion byproducts, and silicate-based ordinary Portland cement. The results showed that the CSA cement matrixes with high stiffness and packing density had the greatest bond strengths for steel and synthetic fibers.

### 2.3. Fiber Content/Group Effect

Zhou and Qiao (2019) [29] showed that the increase in volumetric fiber fraction improves the composite tensile strength, but the rate of improvement is not linear, and it can decrease when using high fiber contents. The authors proposed a model to predict the UHPFRC tensile behavior based on the analytical pullout model of Lee, Kang, and Kim (2010) [36]. The model considers the contribution of the matrix and fibers. The results were validated with direct tension tests. An underestimated response was obtained analytically for low fiber volume, in which the fibers showed better efficiency. Therefore, the increase in fiber content decreases the fiber bond capacity. This phenomenon is attributed to the group effect and the fiber–matrix interfacial weakening due to the interactions between fibers. Most analytical and numerical models do not consider the group effect, but it significantly influences the material response.

Li, Wang, and Backer (1990) [7] observed fiber bundling in concrete reinforced with synthetic fibers. The formation of fiber bundles reduces the contact area interacting with the matrix. Thus, fiber bundles can introduce zones of weakness into the composite as these regions have less resistance. For PVA fibers in SHCC, Yu, Chen, and Leung (2018) [39] found a reduction in bond strength of 30% for 2% of PVA fibers and 40% for 2.5% of PVA fibers. Since there is a low correlation between the single-fiber pullout behavior of aligned fibers and the bending performance, it is difficult to predict the composite behavior using just the single-fiber pullout result. It should be emphasized that numerical/analytical models for SHCC generally do not consider such an effect.

**Figure 5:**
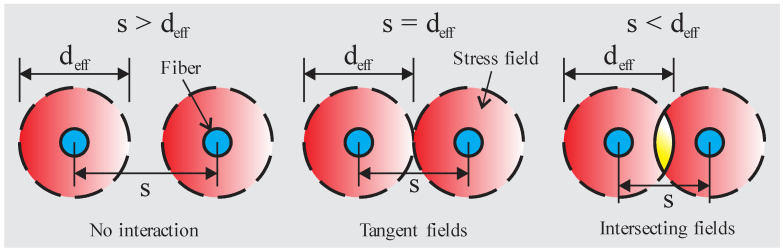
Schematic diagram of the fiber spacing.

Huo et al. (2021) [40] proposed a constitutive model for Fiber-Reinforced Concrete (FRC), considering the interaction between neighboring fibers. The authors suggested that the group effect becomes significant when the spacing between fibers *s* is smaller than an influence diameter *d_eff_*. By analogy to the pile group effect under the negative friction resistance, the following was adopted: *d_eff_* = 6*d_f_* (Figure 5, adapted from Huo et al. (2021) [40]). Based on this theory, the critical fiber volumetric fraction is 4.58%. After this limit, the group effect must be considered. In addition, the authors proposed a coefficient considering the fiber spacing calculated with the ratio between the area of a circle with diameter *s* and the fiber influence (area of a circle with diameter *d_eff_*). This coefficient is given by *η_s_* = *A_s_*/*A_eff_*, where *A_s_* = π(*s*/2)^2^ and *A_eff_* = π(*d_eff_*/2)^2^. The model was implemented via the UMAT subroutine in the Abaqus software by the researchers and was validated with experimental results. The experimental results of Kim and Yoo (2019) [31] indicate a group effect for UHPFRC already with 1% of fiber content. Therefore, further investigations into this topic should be carried out.

Furthermore, no experimental investigations were reported on the influence of matrix reinforcement on group effect, which is expected to occur as fibers surrounding a fiber being pulled provide confinement and can enhance pullout performance, as shown in Benedetty et al. (2021) [41].

### 2.4. Fiber Embedded Length

**Figure 6:**
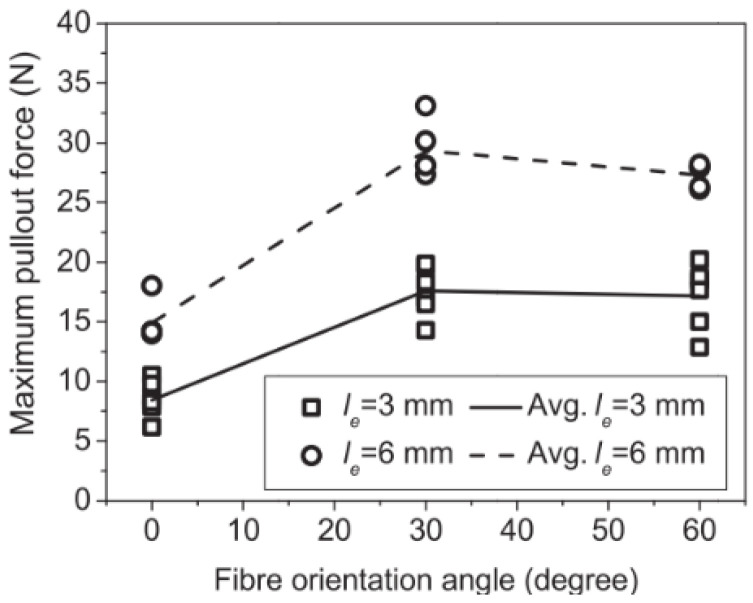
Fiber orientation and embedded length influence the maximum pullout force (Abrishambaf, Pimentel, and Nunes (2019) [17]).

Fibers initially adhere to the matrix and then develop friction during composite straining. The load carried by both mechanisms naturally depends on the embedment length as the fiber load is transferred to the matrix by shear. Such behavior is predominant in straight fibers. Abrishambaf, Pimentel, and Nunes (2019) [17] proposed a constitutive model considering micro- and mesomechanic phenomena for predicting the tensile behavior of UHPFRC. The authors performed the pullout test to obtain average bond strength with single steel fibers with a 0.175 mm diameter and 12 mm length immersed in the UHPC matrix with an embedded length of 3 and 6 mm and inclination of 0°, 30°, and 60°. The maximum load occurred with the embedded length of 6 mm and inclination of 30° (Figure 6, Abrishambaf, Pimentel, and Nunes (2019) [17]). The embedded length is uniformly distributed in the 0 to *l_f_*/2 domain, so the average embedded length is *l_f_*/4. Thus, in the numerical model, the authors adopted the bond strength corresponding to the inclination of 30° and embedded length of *l_f_*/4, with the simplified function *g*(*θ*) =1 for *θ* between 0 and 60°.

**Figure 7:**
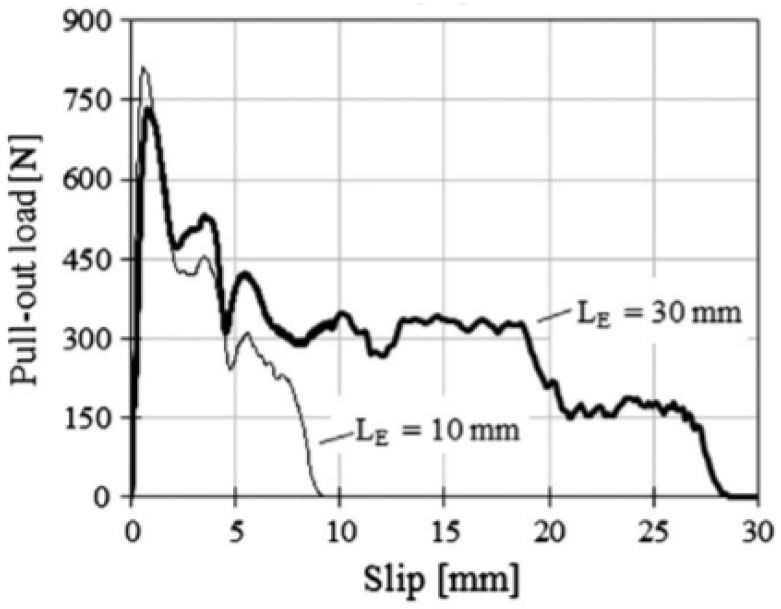
Effect of the fiber embedded length in the pullout load-slip curve (Soetens et al. (2013) [42]).

Soetens et al. (2013) [42] investigated hooked-end fibers with a 0.80 mm diameter and two fiber-embedded lengths (10 and 30 mm). According to the authors, the embedded length has no apparent effect on the pullout response until the hook of the fibers is straightened, as shown in Figure 7 (Soetens et al. (2013) [42]).

### 2.5. Fiber Length and Diameter

Zhou and Qiao (2019) [29] investigated four fiber aspect ratios through analytical models, namely *l_f_/d_f_* of 6/0.16, 13/0.2, 19/0.3, and 25/0.38 mm/mm, resulting in 37.5, 65.0, 63.3, and 65.8 aspect ratios, respectively. The tensile response of the investigated UHPC with 2% steel fibers showed that the higher the aspect ratio, the greater the material tensile strength. Furthermore, the highest fiber aspect ratio results in fewer fibers crossing the crack plane, with less interference between them. On the other hand, the short fiber results in a higher fiber density in the crack plane and, consequently, a higher group effect. Fibers with 13/0.2, 19/0.3, and 25/0.38 mm/mm had similar tensile capacities. However, UHPC with longer fibers showed higher energy absorption due to more effective bridging action, reflected by the strain hardening behavior. Pyo, El-Tawil, and Naaman (2016) [43] evaluated the tensile behavior of UHPFRC at high strain rates. The study tested twisted fibers and two types of straight fibers. The results indicated that the samples with twisted fibers presented better mechanical properties.

Regarding the straight ones, fibers with higher aspect ratios generally showed better mechanical behavior than fibers with lower aspect ratios. For example, samples with fibers of aspect ratio (*l_f_/d_f_)* equal to 125 showed 10%, 40%, and 77% greater postcracking strength, energy absorption capacity, and deformation capacity, respectively, than fibers with *l_f_/d_f_* of 62.5 under impact load. The authors’ results indicate that *l_f_/d_f_* is also critical at high strain rates. Yoo et al. (2017) [44] studied the effect of *l_f_/d_f_* on the flexural behavior of UHPFRC. The study comprised three different *l_f_/d_f_* ratios: fibers designated SS had *d_f_* equal to 0.20 and *l_f_* equal to 13 (*l_f_/d_f_* = 65), those designated SM had *d_f_* equal to 0.20 and *l_f_* equal to 19.5 (*l_f_/d_f_* = 100), and those designated SL had *d_f_* equal to 0.30 and *l_f_* equal to 30 (*l_f_/d_f_* = 100). Fibers with a higher aspect ratio performed better in flexural tests than those with a lower *l_f_/d_f_*. Samples with SM fibers had higher flexural strength than samples with SS fibers. In contrast, the results of the beams with SM and SL fibers were similar since the aspect ratios were similar. The authors performed a cost analysis and pointed out that the total production costs of the material were reduced by up to 32 to 35% when replacing SS fibers with SM or SL fibers. Although studies suggest that fibers with a higher aspect ratio perform better, further studies are necessary to evaluate the isolated effect of the aspect ratio, for example, maintaining the diameter constant and varying the length.

### 2.6. Fiber Orientation

Flow patterns influence fiber orientation in UHPFRC during the fresh state, namely, the fresh-state behavior of the mixture, casting methods, wall effect, mixture pumping, and applied magnetic field (Huang, Gao and Teng, 2021 [45]; Pae et al., 2021 [46]; Zhou and Uchida, 2017 [47]; Švec et al., 2014 [48]). The material mechanical performance can be significantly improved with fiber alignment when the fibers are preferably aligned in the principal stresses (Duque and Graybeal, 2017 [4]; Bastien-Masse, Denarié and Brühwiler, 2016 [49]; Kang and Kim, 2011 [50]). Despite the significant improvement, it is essential to emphasize that fiber orientation during pouring can result in anisotropy in UHPFRC performance.

**Figure 8:**
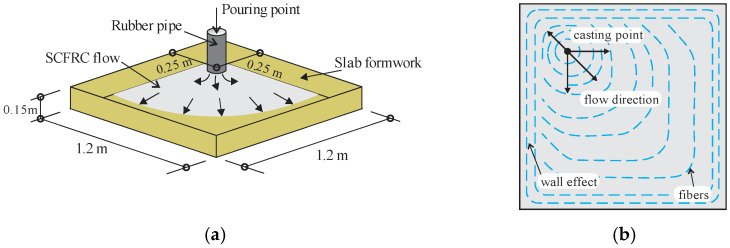
(**a**) Slab casting. (**b**) Comparison of the simulated fiber orientations (black stroke) with the computed tomography results (red stroke).

Švec et al. (2014) [48] studied the influence of surface roughness of the formwork on the steel fiber orientation and the resulting mechanical response of the structural components made of self-compacting concrete. The casting process was conducted from a rubber pipe inlet positioned near one of the corners of the slab (Figure 8a, Švec et al. (2014) [48]). Observations of fiber orientation indicated that fibers tended to orient according to the flow direction during casting. However, fiber orientation exhibits greater randomness near the rough surfaces. Figure 8b (adapted from Švec et al. (2014) [48]) shows the fiber orientation obtained by tomography and computational modeling. The authors highlight that fiber orientation and structural element properties depend highly on material rheology and casting method. Furthermore, the macroscopic properties at the slab center and periphery layers differ due to the wall effect. Therefore, the variation in surface roughness combined with the wall effect influences the uncertainties in the behavior of structures made of fiber-reinforced self-compacting concrete.

**Figure 9:**
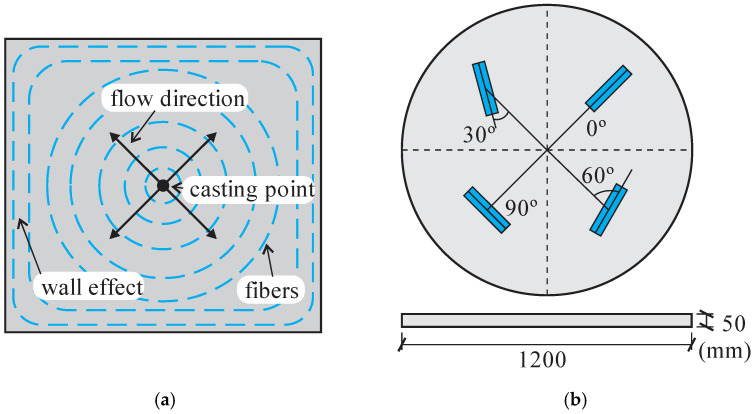
(**a**) Cutting locations of specimens in a circular UHPFRC panel. (**b**) Fiber alignment on a plate.

Zhou and Uchida (2017) [18] evaluated the influence of UHPFRC fresh state properties on the fiber alignment and its mechanical behavior. In a slab sample, fiber orientation varied along the specimen height. Fibers were aligned as circles from the casting position in the upper half and obliquely upward at the bottom, as shown in Figure 9a (adapted from Zhou and Uchida (2017) [18]). The fibers were oriented parallel to the longitudinal direction at the slab bottom face, mainly due to the shear force from the interaction with the formwork. The results indicated that flowability dictates the final fiber orientation in parts close to the formwork surfaces. More flowable UHPFRC results in more fibers oriented parallel to the longitudinal direction of slabs. In addition, flowability and pouring time influence the hardened properties of UHPFRC significantly. After initial cracking, bending capacity exhibited linear relation with the number of fibers in the fracture planes. Zhou and Uchida (2017) [18] studied the relationship between fiber orientation and the postcracking behavior of UHPFRC. A plate was cast with concrete poured at the formwork center. Samples were extracted with angles of 0°, 30°, 60°, and 90° relative to panel diametrical direction (Figure 9b, adapted from Zhou and Uchida (2017) [18]). The mechanical behavior of the samples was evaluated with three-point bending tests. Fiber orientation was obtained with image analysis and 3D visualization from x-ray CT. The postcracking flexural strengths of specimens cut at 60°, 30°, and 0° were 80, 40, and 10% smaller than those cut at 90°, indicating dependency on the fiber contribution close to the fracture surfaces.

Moreover, the fiber orientation can be evaluated by various tests such as image analysis, CT scan, translucent, viscous fluid, and electrical or magnetic methods (Huang, Gao and Teng, 2021 [45]). In addition, several parameters can describe the fiber orientation. For example, fibers counted in 1 mm^2^ area (*F_n_*) are given by Equation (4):

(4) $$F_{n}=\frac{n_{f}}{A}$$

where *n_f_* is the fibers counted in the cut plane, and *A* is the image area.

When using image analysis, the angle between the fiber and the direction normal to the cutting plane *θ* can be calculated from Equation (5) when the fiber is projected into the cutting plane:

(5) $$\theta =\arccos(d_{f}/l)$$

Cutting a section for analysis, the fiber projection on the cutting plane is a circle or ellipse, where *d_f_* and *l* are the smallest and largest axis of the fiber ellipse. Note that for *d_f_/l* equal one, the fiber section is circular, therefore, the fiber axis is normal to the view plane. Conversely, when *d_f_/l* tends to zero, there is an indication that *l* is much larger than *d_f_*, i.e., the fiber is oriented perpendicularly to the cutting plane.

An alternative definition is the orientation coefficient *η_θ_* (Wille, Tue and Parra-Montesinos, 2014 [51]), which can be determined as the mean of the cosine of the orientation angle of the fibers that cross the cut section, according to Equation (6):

(6) $$\eta_{\theta }=\frac{1}{n_{f}}\sum_{i=1}^{n_{f}}\cos\theta_{i}$$

where *n_f_* is the number of fibers in the view plane, and *θ* is the fiber axis angle and the plane’s normal direction. The equation indicates that fibers are aligned in the normal direction when *η_θ_* = 1 and perpendicularly aligned when *η_θ_* = 0.

Table 1 shows the orientation coefficient obtained from tensile samples. The loading direction of the model determined the orientation coefficient. These results illustrate the influence of the pouring method on the orientation coefficient and, consequently, on the hardened behavior of the composite. Research indicates that *η_θ_* equals 1, 2/π, and 0.5 for uniformly random 1D, 2D, and 3D distribution of fibers (Švec et al., 2014 [48]). Note that most samples show a tendency to preferential alignment with one direction. Furthermore, it is possible to achieve a high orientation coefficient using techniques such as the electromagnetic field; see the results of Abrishambaf, Pimentel, and Nunes (2019) [17].

**Figure 10:**
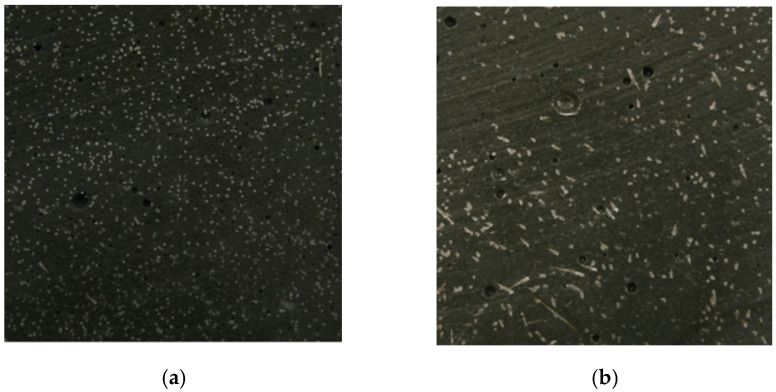
Distribution and orientation of the fibers: (**a**) PL samples, (**b**) TL samples (Kang and Kim (2011) [50]).

Kang and Kim (2011) [50] investigated the influence of the placement direction, the material tensile behavior, and the fiber distribution. The specimens labeled PL were produced with the concrete cast parallel to the tensile stress direction. In contrast, the models marked TL were made with the concrete cast transversal to the tensile stress direction. As a result, the fibers in the PL samples are more aligned normally to the cutting plane than in the TL samples (Figure 10, Kang and Kim (2011) [50]). The direct tensile tests showed that the first crack occurred with 10.93 MPa for the PL samples and 9.96 MPa for the TL samples. Furthermore, the maximum stress achieved was 16.05 MPa for the PL samples and 11.80 MPa for the TL samples. A favorable fiber distribution and orientation made it possible to increase the stress corresponding to the formation of the first crack by approximately 10% and the maximum stress by almost 40%.

### 2.7. Fiber Distribution Effect

**Figure 11:**
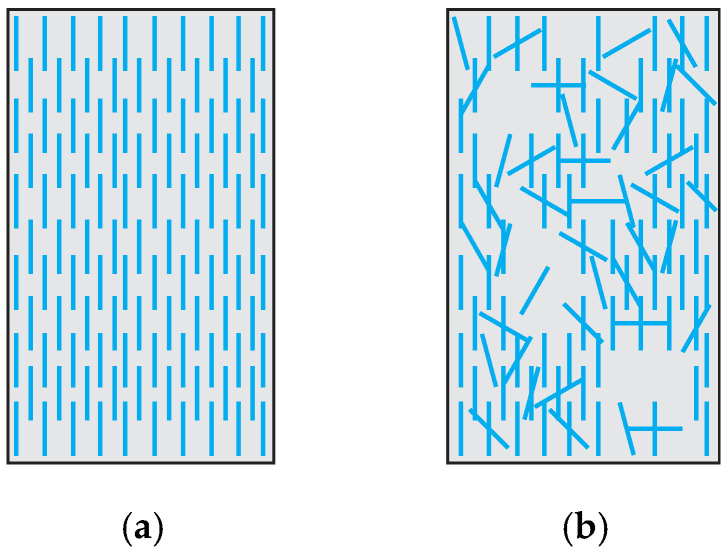
Factors representing fiber dispersion: (**a**) *μ*_2_ = 1.0, (**b**) *μ*_2_ < 1.0.

The mixing and consolidation process dramatically influences the uniformity of fiber distribution. Stereological models can analyze fiber distribution (Bentur and Mindess, 2006 [52]). Shen and Brühwiler (2020) [53] introduced a uniformity factor *μ*_2_ to consider the local fiber distribution in UHPFRC elements. This factor is a scalar indicator of the degree of uniformity in the local fiber distribution. *μ*_2_ = 1.0 corresponds to a homogeneous material, that is, fibers are spaced and oriented equally, while *μ*_2_ < 1.0 corresponds to anisotropic behavior, as depicted in Figure 11. The influence of *μ*_2_ on the tensile response was investigated through an experimental campaign. The authors concluded that fiber distribution is an essential factor in tensile behavior. The local distribution of fibers governs the strain-hardening response of SHCC. Some regions with nonuniform fiber dispersion become critical for the entire sample, compared to the remaining parts that would develop the hardening capacity.

### 2.8. Fiber Hybridization

The typical fibers used in UHPFRC are high-strength, straight steel fibers, usually coated to enhance friction and protect against corrosion. However, hybrid solutions are being investigated to improve the composite performance due to synergistic effects (Banthia and Sappakittipakorn, 2007 [54]; Banthia et al. 2014 [55]). Different fibers (in diameter and length) can reinforce cracking at different scales. For example, combining the different sizes of steel fibers (Yoo, Kim, and Park, 2017 [56]; Chun and Yoo, 2019 [57]), different types of fibers, and multiwalled carbon nanotubes can reinforce different crack sizes and enhance strength and toughness. Yu, Chen, and Leung (2018) [39] studied the crack-bridging relations of SHCC, combining PVA and steel fibers with a total volume fraction of 2.5%. According to Equation (7), the numerical model considered the superposition of the contribution of the different components (*σ_m_*—matrix stress, *σ_Steel_*—steel stress, and *σ_PVA_*—PVA stress). The authors found a positive synergetic effect at the single-crack level under uniaxial tension.

(7) $$\sigma =\sigma_{m}+\sigma_{PVA}+\sigma_{Steel}$$

Furthermore, fiber hybridization can improve the performance of the composite under high-temperature conditions. Mindeguia et al. (2010) [58] investigated such behavior using a device to measure the temperature of concrete specimens, pore vapor pressure, and mass loss. The authors tested five concrete dosages, maintaining aggregate volume constant but varying water/cement ratios. The research aimed to understand concrete’s behavior at high temperatures and its correlation to spalling. Spalling has two main mechanisms: (1) A thermomechanical process involving high-temperature variables and inducing high compressive stresses in the concrete. These stresses can exceed the strength of the concrete and cause spalling. (2) A thermo-hygral process, which is due to the movement of fluids present in the concrete due to pressure gradients and molar concentration. Water vapor begins to condense and cause pressure on the pores, possibly exceeding the tensile strength and initiating fragmentation (Mindeguia et al., 2010 [58]). The results showed that (1) the low concrete compaction (high *w*/*c* ratio) induces greater permeability to fluids and facilitates water escape; (2) low permeability involves high pore pressure accumulation, so the lower the *w*/*c* ratio, the higher the pore pressure; (3) a dense matrix results in a higher temperature for vaporization; and (4) thermal flow is quite similar among the five concrete mixes. These findings explain why UHPC, concrete with a low *w*/*c* ratio, is more susceptible to spalling than conventional concrete. Research indicates that PP fibers allow the prevention of explosive spalling (Li and Zhang, 2021 [59]; Li, Tang and Yan, 2019 [60]; Ding et al., 2016 [61]; Bangi and Horiguchi, 2012 [62]). Li and Zhang (2021) [59] evaluated the behavior of UHPC without fibers, only with PP fibers, and with steel and PP fibers. The authors observed that the material’s mechanical properties were not changed by adding PP fibers because these fibers have low strength and a micrometrical diameter combined with the small strength and stiffness of the synthetic material. Therefore, fibers tend to break due to the high bond of the UHPC without adding strength or deformability contributions at a hardened state. It can be said that the primary role of PP fibers is to control shrinkage. 

**Figure 12:**
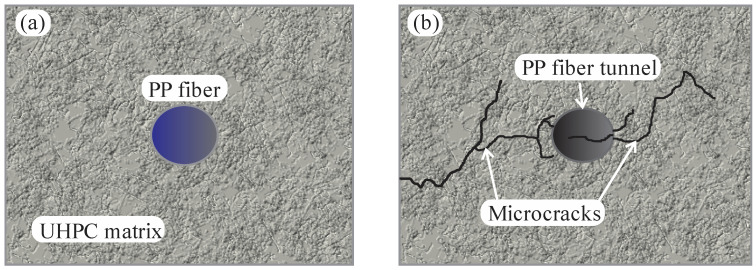
PP fibers in UHPC: (**a**) before and (**b**) after exposure to 200 °C.

In contrast, the compressive tensile strength and elasticity modulus were increased by using steel fibers. Tests have shown that the simultaneous inclusion of PP and steel fibers can prevent explosive spalling. Li, Tang, and Yan (2019) [60] find that explosive spalling was prevented entirely in UHPC when using hybrid PP and steel fibers at low fiber dosage. The low melting temperature of synthetic fibers forms a network of fiber tunnels, increasing the permeability significantly for water vapor to leave the matrix. Melting of PP fibers starts at 150 °C and is finished at 176 °C. Figure 12 (adapted from Li, Tang and Yan (2019 [60]) shows schematically what was observed in MEV images of PP fibers in UHPC before and after heating at 200 °C. The fibers left a network of tunnels and microcracks after the thermal expansion of both synthetic and steel fibers, implying a synergistic effect can be attributed. 

### 2.9. Size Effect

The size effect occurs in concrete due to the available area to form the fracture process zone (Kwon, Zhao and Shah, 2008 [63]). However, adding fibers reduces the size effect due to the ductility provided (Nguyen et al., 2014 [64]). Mahmud, Yang, and Hassan (2013) [65] tested the flexural strength of notched UHPFRC beams under three-point bending tests to investigate the size effect. The authors found that the nominal strength was less influenced by the size effect due to the high ductility of UHPFRC. Nguyen et al. (2013) [66] investigated the bending strength of Ultrahigh-Performance Hybrid Fiber-Reinforced Concrete (UHP-HFRC) to understand the size effect. Four-point bending tests were performed considering three specimen sizes. In addition, two mixtures were evaluated. UHP-HFRC1 was made up of 0.5% smooth steel microfiber (SS-fiber) and 1% twisted steel macrofiber (T-fiber) by volume, while UHP-HFRC2 had 1.0% T- fiber and 1.0% SS-fiber. The results showed that bending strength, deflection, and energy absorption capacity were affected by the size effect significantly. According to Weibull’s theory regarding the size effect, the larger specimen is, the more elements in the chain exist, so smaller samples are less prone to failure.

In contrast, the Bazant theory for size effect states that the larger specimens release more energy into the crack front than the smaller ones. Therefore, the decrease in sample size increases the flexural strength, deflection, and energy absorption capacity of UHP-HFRC. Yoo and Banthia (2016) [67] suggested that fiber distribution characteristic is the main factor for the size effect in UHPFRC beams, i.e., UHPFRC beams containing 2% steel fibers with uniform fiber distribution have an insignificant size effect on the flexural strength. Nguyen et al. (2014) [64] studied the size effect on UHPFRC’s tensile behavior composed of 1% macrotwisted and 1% microsmooth steel fibers by volume. The authors concluded that strain capacity, energy absorption capacity, and crack spacing of UHPFRC were susceptible to parameters such as gauge length, section area, volume, and thickness, while the postcracking strength was not. Overall, the size effect involves several phenomena that reflect on the mechanical behavior of the UHPFRC, such as the influence on fiber orientation and material shrinkage, which is related to the stress perpendicular to the fibers and, consequently, the frictional strength. The investigation of each isolated effect is complex but necessary to understand the size effect fully. Therefore, further research is required, and it should be highlighted that no results were found on the size effect on the direct tensile strength of SHCC.

### 2.10. Preparation of Fiber-Reinforced Compositions

Most Strain-Hardening Cement Composites (SHCCs) are designed to be self-consolidating mixtures. The advantage is better matrix homogenization and fiber dispersion. Using chemical admixtures as plasticizers is a common strategy, which can be combined with sophisticated methods for self-consolidating fiber-reinforced concretes as the liquefaction approach, based on particle packing techniques, according to Lepech and Li (2008) [68]. The modified Andreasen and Andersen model is used. The technique allows the design of high packing density mixtures with low water-to-cement ratio, and the self-consolidating behavior arises from the high pore pressure by the energy provided by the mixer, causing the liquefaction phenomena. The casting process also requires care for the finished structural component for developing the designed performance, aiming to minimize mechanical property variability. According to Li (2019) [8], plastic viscosity control can avoid poor fiber dispersion, which can be helped by optimizing the mixing sequence. 

In the context of dosage and technology for preparing fiber-reinforced composites, Ruslan, Ruslan, and Evgenij (2022) [69] studied the effect of metal and polypropylene fiber on technological and physical–mechanical properties of activated cement compositions. The research investigated the effect of various types of fibers on the rheological properties of concrete mixes and the physical and mechanical properties of self-compacting concrete and mortars obtained by activating Portland cement in a vortex layer device (VLD). The authors analyzed dispersed reinforcement of fibrous concrete based on the Schklowsky–De Zhen theory. Through the experiment’s mathematical planning method, the optimal content of metal and polypropylene fiber was established: polydisperse reinforcement with metallic fiber, 0.5%, and polypropylene, 0.45% in volume. As one of the results of the study, a self-sealing fibrous concrete was developed, obtained by activating the binder in VLD, with low shrinkage (0.2 mm/m), porosity (7.4%), and water absorption (up to 1.51%); high resistance to frost and water; and high resistance to aggressive media. The study indicates the importance of the technology for preparing and optimizing fiber-reinforced compositions and the effect of the type of fiber on the material’s mechanical response. Therefore, such factors must also be considered when predicting the SHCC tensile behavior.

## 3. Analytical Modeling Methodologies

### 3.1. Micromechanical Models

**Figure 13:**
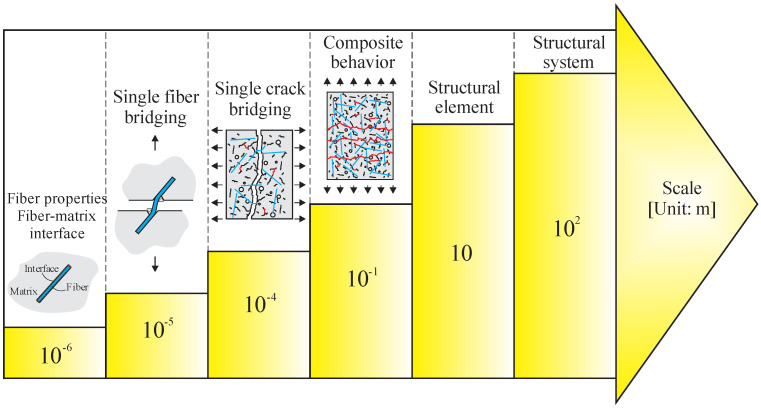
Scaling up modeling of FRC.

Analytical models based on micromechanical phenomena have been developed and implemented to simulate fiber-reinforced concrete (Abrishambaf, Pimentel and Nunes, 2019 [17]; Lee, Cho and Vecchio, 2011 [26]; Li, 1992 [27]). These models start taking parameters from a microscopic view, studying the phases (fiber, matrix, and fiber–matrix interface properties) on a macroscopic scale, as illustrated in Figure 13 (Yao and Leung (2020) [70]). Li (2019) [8] highlights that the micromechanical model considers microscale phenomena, such as interfacial slippage with chemical or adhesive debonding and microcrack opening, and aspects at higher scales, such as fiber length. In addition, matrix properties and interfacial parameters indirectly consider other features such as composite composition, fiber surface treatment, and porosity. In such methodologies, fiber distribution and orientation are considered statistically. However, fibers are not explicitly considered, so it presents advantages relative to computational cost compared to multiphase models being more prominent solutions for structural analysis.

### 3.2. Stress-Crack Opening Curve Based on Micromechanical

**Figure 14:**
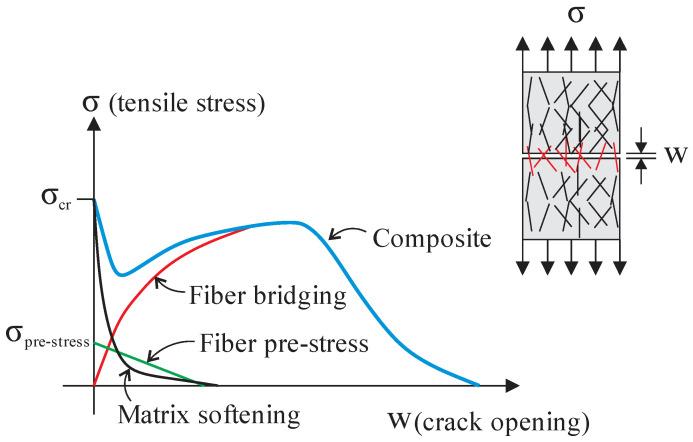
Typical tensile stress-crack opening curve.

Li (1992) [27] proposed the stress-crack opening curve considering the fiber bridging mechanism for randomly oriented fibers in a cementitious matrix. The postcracking response of the material was based on micromechanical phenomena, such as fiber–matrix interfacial behavior. The theoretical results were compared with concrete reinforced with steel or synthetic fibers tests, showing good agreement. The stress-crack opening curve is separated into prepeak and postpeak parts. The prepeak is known as the debonding phase, or according to Li (1992) [27], it should be understood as the frictional slip activation. After complete fiber debonding, fiber pullout takes place. On UHPC, the behavior is similar. Linear elastic stress transfer occurs before the initiation of the first crack. Subsequently, microcrack formation and partial debonding happen across the fiber–matrix interface. Finally, the fiber debonds totally from the matrix, and friction is the primary mechanism for stress transfer. The fiber controls the postcracking behavior of UHPC in tension (Zhou and Qiao, 2019 [29]). The material tensile stress-crack opening behavior can be obtained analytically by adding three phenomenological parts: the matrix softening stress, the fiber prestress, and the fiber bridging stress in cracks (see Figure 14).

#### 3.2.1. Matrix Stress

The UHPC tensile behavior is brittle, i.e., there is a sudden decrease in load after the peak. Therefore, the softening behavior of the cementitious matrix can be determined by the matrix cracking strength (*f_mt_*) and fracture energy (*G_Fm_*). The tensile response can be considered, for example, as a bilinear or exponential curve. Abrishambaf, Pimentel, and Nunes (2019) [17] adopted an exponential stress-crack opening curve (*σ_mt_*), expressed as Equation (8):

(8) $$\sigma_{mt}=f_{mt}\exp(-f_{mt}w/G_{Fm})$$

#### 3.2.2. Fiber Prestress

The fibers are also deformed during the composite straining in the elastic regime. Therefore, fiber prestress (*σ_pre_*) occurs before matrix cracking, which is gradually relieved. A linear variation is assumed for the fiber prestress, which becomes null after the complete debonding of the fiber, as shown by Equation (9):

(9) $$\sigma_{pre}=f_{mt}\gamma (w_{deb}-w)/w_{deb}\geq 0$$

The coefficient *γ* ranges between 0.05 and 0.17 for UHPFRC, with fiber volume fractions between 0.02 and 0.04. Matrix Young’s Modulus typically ranges between *E_m_* ≈ 40 to 55 GPa, and the steel fiber modulus of elasticity is in the range *E_f_* ≈ 200 to 210 GPa. $w_{deb}=\tau l_{f}^{2}/E_{f}d_{f}$ is the crack width that represents the beginning of the pullout, where *τ* is the interfacial bond strength, *l_f_* is the fiber length, and *d_f_* is the fiber diameter.

#### 3.2.3. Fiber Bridging Action

After matrix cracking, the stress-displacement curve is governed by fiber bridging (Li, 1992 [27]). Li, Wang, and Backer et al. (1991) [71] showed that stresses at a crack could be estimated by integrating the contribution of individual fibers, using Equation (10):

(10) $$\sigma_{f}(w)=\frac{V_{f}}{A_{f}}\int_{\theta_{0}}^{\theta_{1}}\int_{z=0}^{(L_{f}/2)\cos\theta }P(w,l_{e})g(\theta )p(\theta )p(z)dzd\theta$$

where *P*(*w*,*l_e_*) is an analytical pullout function of a single fiber normal to crack plane and with embedment length *l_e_*; *p*(*θ*) represents the fiber inclination angle randomness; *p*(*z*) is the centroidal fiber location randomness to the crack face; and *z* is the distance between the fiber centroid and the crack plane (considering that *z* varies between 0 and *l_f_*/2, resulting in *p*(*z*) = 2/*l_f_*). The integration limits between *θ*_0_ and *θ*_1_ is the range of the fiber inclination. The inclined fiber bridging force *p*(*θ*) is correlated to the aligned fiber pullout *P*(*w*,*l_e_*) via the term *g*(*θ*), i.e., *p*(*θ*)= *P*(*w*,*l_e_*) *g*(*θ*). The higher orientation of the fibers to the crack surface, the higher the resulting strength, while fibers with a small inclination to the crack surface lose bridging capacity in these areas (Zhou and Uchida, 2017 [47]).

Li (1992) [27] solved the integral of Equation (10), considering the random distribution of fibers, *g*(*θ*) equals *e^f^^θ^* and *P*(*w*,*l_e_*), given by Equation (2). Similar expressions are still used to predict the tensile behavior of UHPFRC (Abrishambaf, Pimentel and Nunes, 2019 [17]). Recently, Ribeiro, Krahl, and Carrazedo (2022) [34] solved the integral for the analytical model *P*(*w*,*l_e_*) that includes the interfacial parameter *β*, the group effect coefficient *ξ*, and any orientation. Hence, the fiber contribution to the composite strength can be calculated from Equation (11):

(11a) $$\sigma_{f}(\bar{w})=\frac{V_{f}L_{f}\xi \tau }{2d_{f}}\cdot \left[4{\left(\frac{\bar{w}}{\bar{w^{\prime }}}\right)}^{1/2}-2\left(\frac{\bar{w}}{\bar{w^{\prime }}}\right)\right]\int_{0}^{\pi /2}p(\theta )g(\theta )\cos(\theta )d\theta ,\;\bar{w}\leq \bar{w^{\prime }}$$

(11b) $$\sigma_{f}(\bar{w})=\frac{V_{f}L_{f}\xi \tau }{2d_{f}}\cdot \left[2{\left(1-\bar{w}\right)}^{2}+\frac{\beta L_{f}}{d_{f}}\left(1-\bar{w}\right)\left(\bar{w}-{\bar{w}}^{2}\right)\right]\int_{0}^{\pi /2}p(\theta )g(\theta )\cos(\theta )d\theta ,\;\bar{w}>\bar{w^{\prime }}$$

where, $\bar{w^{\prime }}=2\xi \tau L_{f}/[E_{f}d_{f}(1+\eta )]$ and $\bar{w}=w/(l_{f}/2)$.

Table 2 presents some results of $\int_{0}^{\pi /2}p(\theta )g(\theta )\cos(\theta )d\theta$, representing the fiber efficiency in delaying the crack propagation.

#### 3.2.4. Modeling Multiple Cracking

**Figure 15:**
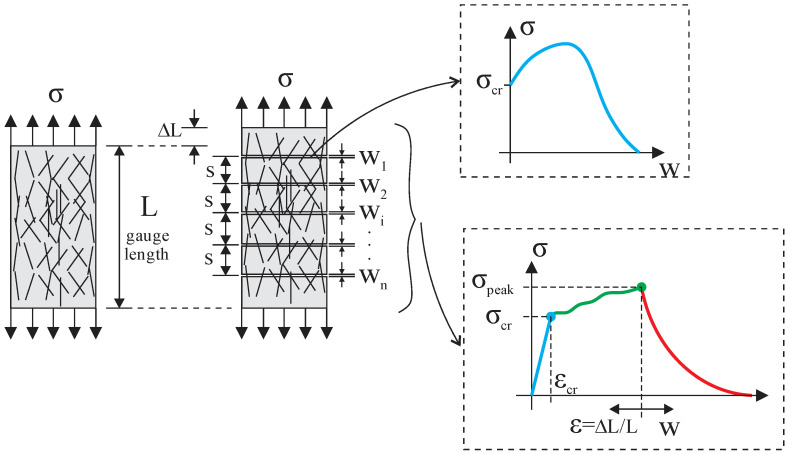
Crack bridging and multiple cracking development.

Composites with strain-hardening behavior can develop multiple cracks under tensile stress as loading increases. The increase occurs because the bridging fibers can carry more stress than the matrix, as shown in Figure 15. Therefore, the composite tensile strength is reached when the crack bridging at the weakest crack achieves its maximum value. Furthermore, the fiber load transfer mechanism promotes successive neighbor cracks during the process, and the crack spacing (*s*) and width (*w*) govern the strain at peak load. The micromechanics-based design considers the mentioned processes and can be a powerful tool for tuning component performance, selecting ingredients for mixing optimization, and developing new strain-hardening materials.

The deformation of the composite can be calculated, assuming a crack spacing model and the stress-crack opening curve stress–strain (*σ*-*w*) obtained from Equation (11). The total deformation *ε* is then obtained with Equation (12):

(12) $$\varepsilon =\varepsilon^{el}+\varepsilon^{cr}=\frac{\sigma }{E}+\frac{\sum w_{i}}{L}$$

Σ*w_i_* is the sum of all crack openings developed until the applied stress, as depicted in Figure 15. Some studies consider only the crack opening to determine the total strain, such as that by Lu, Leung, and Li (2017) [72].

Recently, several models have been proposed for predicting strain-hardening behavior. Most of them are based on the model developed by Aveston, Kelly, and Copper (1971) [73], the ACK model. The initial idea was to determine the distance necessary to transfer the bridging forces in the fibers at cracks to the matrix, considering aligned fibers. The cracking strength was constant, resulting in all cracks forming simultaneously. If the distance for stress transfer is *s*, and accounting for constant shear stress at the interface *τ*, the equilibrium resulting in the full cracked composite is equal to $2\pi r\tau_{fu}sN$ or $2\tau_{fu}sV_{f}/r$, in which *r* is the fiber radius, and *N* is the number of fibers. Then, this force is taken equal to the cracking strength of the matrix (*σ_mu_* × *V_mu_*), which means that at a distance of *s*, the total bridging forces were transferred to the matrix. Thus, the crack spacing *s*_1_ is calculated as follows:

(13) $$s_{1}=\frac{V_{m}}{V_{f}}\cdot \frac{\sigma_{mu}r}{2\tau_{fu}}$$

Where *V_m_* and *V_f_* are the matrix and fiber volume fractions, respectively, and *σ_mu_*, *r*, *τ_fu_* are the matrix strength, fiber radius, and average bond strength, respectively. Due to the hypothesis of constant shear stress at the fiber interface, the strains and stress transferred from the crack to the matrix are assumed linear. Thus, the stress sustained by the composite is assumed constant (only frictional slip occurs) until segments of *s* or 2*s* length separate the entire matrix. This trend occurs due to the adopted constant matrix cracking strength, which is known today as inconsistent with the experimental evidence. Aveston and Kelly (1973) [74] added the fiber orientation coefficient *η_θ_* in the ACK model by multiplying Equation (13) by 1/*η_θ_*. In the case of random distribution *η_θ_* = 0.5, Equation (13) is multiplied by 2. Abrishambaf, Pimentel, and Nunes (2019) [17] used similar approaches to simulate changes in behavior due to the fiber orientation of Ultrahigh-Performance Concretes.

Equation (13) predicts the cracking spacing for discontinuously reinforced matrices with an error of less than 15%. The fundamental difference is that composites with short fibers present strain-softening after multiple cracking, as pullout concentrates on the localized crack after the peak in the *σ*-*w* curve. Aveston and Kelly (1973) [74] presented another equation for the crack spacing *s*_2_ for discontinuous fibers, accounting for the influence of the length of discrete fibers, Equation (14):

(14) $$s_{2}=\frac{L_{f}-\sqrt{L_{f}^{2}-2\pi L_{f}s_{1}}}{2}$$

where *L_f_* is the fiber length, and *s*_1_ is given by Equation (13). Wu and Li (1992) [75] extended the model from Equation (14) to account for the impact of the snubbing effect (concentrated stresses at the end of fiber tunnel for an inclined fiber) on composite crack spacing *s*_3_, resulting in

(15) $$s_{3}=\frac{L_{f}-\sqrt{L_{f}^{2}-4\sigma_{mu}V_{m}\;r/g\tau V_{f}}}{2}$$

where *g* is the snubbing factor (Equation (16)), and *f* is an empirical value:

(16) $$g=2\frac{e^{f\pi /2}}{f^{2}+4}$$

Recently, more advanced theories were proposed considering composite property variabilities. The models account for variations such as fiber content, matrix strength, orientation, strength, and different hypotheses for the stiffness of the bridging fibers. Such factors reflect the nonuniform properties of Short Fiber Cement-Based Composites, typically influenced by mix design and fresh state flowability, production processes, casting methods, curing procedures, etc.

**Figure 16:**
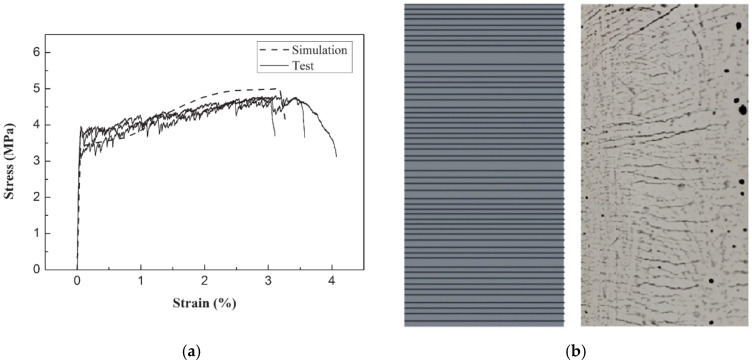
Comparisons between simulated and experimental results of (**a**) stress–strain behavior and (**b**) cracking pattern (Lu and Leung (2016) [76]).

Therefore, considering variation in mechanical properties implies nonuniform crack spacing, which agrees with actual experimental evidence. Lu and Leung (2016) [76] proposed a model accounting for matrix strength variation from such observations. The model checks the crack after each stress increment. When crack strength is reached in the weakest section, the damage is recorded and continues to occur with load increase if the material has strain hardening behavior, generating multiple cracking. The increments range from the cracking strength to the maximum bridging stress at the weakest section. The methodology divided the tensile specimen into several elements, smaller than the crack spacing *s*_3_, Equation (15). Then, a random strength is attributed for each element based on the two-parameter Weibull distribution with shape and scale parameters of 1.1 and 2.4, respectively, as in Wu and Li (1995) [77]. Figure 16a,b (Lu and Leung (2016) [76]) present the comparison between the prediction and experimental stress–strain results and cracking pattern, respectively, for an Engineered Cementitious Composite (ECC) with 2% of PVA fibers. The authors did not consider fiber volume variation, arguing that accounting for such a hypothesis would reduce the number of cracks and, consequently, the composite ductility.

Lu, Leung, and Li (2017) [72] changed the matrix strength variation hypothesis by discussing that the random approach is inconsistent with experimental observations; there is a pattern as such property varies. Accordingly, there is a higher probability that neighbor sections had similar strengths. To represent such a trend, they considered Kabele and Stemberk’s (2005) [78] approach to represent matrix defects (spherical penny-shaped flaws) with a normal distribution function and random locations within the gauge length. The method considers that the same flaw can occupy different neighbor segments, establishing continuity of matrix strength variation. The matrix strength was calculated based on the part of the flaw inside each segment. The specimen length was divided into 200 segments with 0.02 mm for modeling. Another hypothesis was that the stress in the crack face does not start from zero due to the inclined fibers’ pulley effect (snubbing).

**Figure 17:**
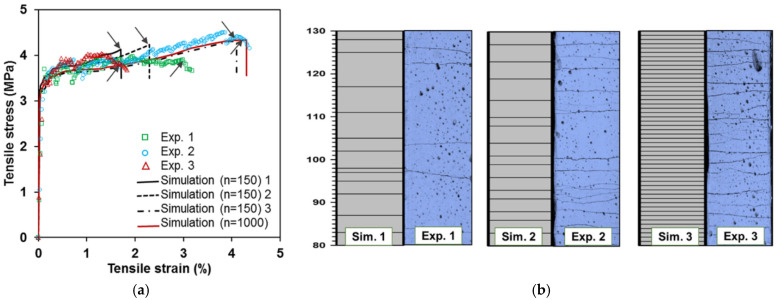
Experimental e predicted variability on crack saturation on ECC: (**a**) stress–strain behavior and (**b**) cracking pattern (Li, Weng and Yang (2019 [79]).

Li, Weng, and Yang (2019) [79] developed a micromechanics stochastic model. The authors considered heterogeneities in fiber, matrix, and interfacial properties, resulting in different fiber-bridging properties for all formed cracks. Random micromechanical variables were assigned for each segment of the divided gauge length. The various properties followed a normal distribution, except the matrix cracking strength, which took a Weibull function. The sample was tested with 150 and 1000 segments for a gauge length of 150 mm, resulting in slight differences. Crack width and spacing varied, implying differences at the end of multiple cracking stages (crack localization) in the simulations, as shown in Figure 17 (Li, Weng and Yang (2019 [79]). However, the research found log-normal and Weibull’s distributions for crack width and spacing, which agree with literature experiments. Moreover, statistical analysis showed that the variations of fiber strength and content are the most sensitive parameters in changing the strain capacity of the composite.

**Figure 18:**
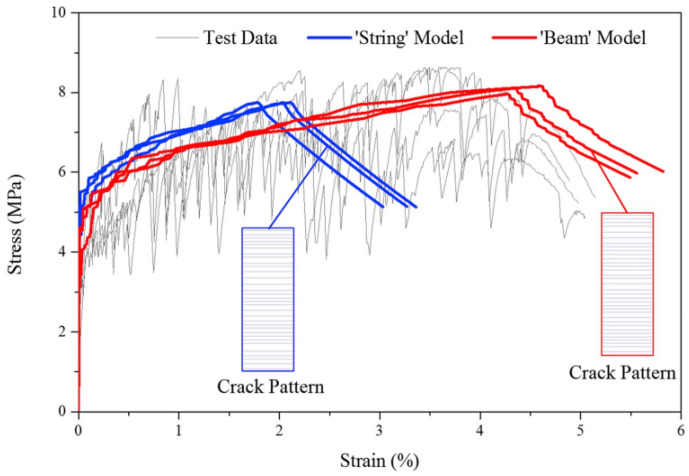
Comparison between prediction models considering fibers as beams and strings (Yao and Leung (2020) [70]).

Yao and Leung (2020) [70] proposed a procedure considering the variation of fiber volume fraction along the sample and the bridging fibers behaving as beams instead of trusses. For the last hypothesis, the argument was that fibers with an elastic modulus of the same order or greater than the matrix stiffness control the crack width and spacing due to the flexural and axial fiber stiffness. Such change predicted higher strain capacities and maximum bridging loads better than models with strings, as depicted in Figure 18 (Yao and Leung (2020) [70]). The developed approach also eliminates the empirical snubbing coefficient *f* in Equation (16). The authors divided the tensile sample of 80 mm into segments with lengths lesser than the predicted crack spacing *s*_2_, Equation (14), to account for matrix strength and fiber content stochastic variations. The segments were 0.05 mm long, as for Lu and Leung (2016) [76], and *s*_2_ was 1.28 mm. Then, *V_f_* and matrix strength were randomly generated and assigned to segments following the Weibull distribution. For fiber content, the scale parameter was *λ* = 2, and the shape parameter was *k* = 40. For matrix strength, *λ* = 6, and *k* = 10. The segments with the smallest *V_f_* controlled the crack localization, as the maximum bridging stresses were smaller than the others. The authors also stated that fiber rupture was still not accounted for in determining the cracking spacing *s*.

The mentioned approaches have the appeal of using the micro- and mesomechanisms observed experimentally. However, it is worth mentioning that there are other alternative methodologies for predicting the mechanical behavior of SHCC, namely, multiphase modeling, in which the fibers are simulated discreetly and immersed in the cementitious matrix (Bitencourt Jr et al., 2019 [20], Qsymah, 2016 [21]; Cunha, Barros and Sena-Cruz, 2011 [22]); inverse analysis, in which the stress–strain relationship of the material is obtained by inverse analysis techniques based on experimental responses (Kang et al., 2010 [80]; Baby et al., 2013 [81], Stephen et al., 2019 [82]); use of different types of machine learning such as artificial neural network (ANN), support vector regression (SVR), classification and regression tree (CART), and gradient boosting tree (GBoost) (Guo et al., 2021 [83]; Abellán-García and Guzmán-Guzmán, 2021 [84], Marani, Jamali, Nehdi, 2020 [85]); and techniques based on the theory of homogenization with the development of multiscale models (Yu et al., 2020 [86]). 

## 4. Current Challenges and Future Research Needs 

The prediction methods for the tensile response of SHCC have advanced significantly since the first studies (Aveston, Kelly and Copper, 1971 [73]; Wu and Li, 1997 [75]). Recently, some experimental evidence that influences the behavior has been found, such as the fiber distribution effect (Shen and Brühwiler, 2020 [53]). In addition, other parameters already known that are not considered in the models, such as the scale effect (Rossi et al. 1994 [87]), are essential for the transition of material behavior to structural performance.

It should be mentioned that the micromechanical models are of general use in developing and predicting the behavior of any FRCC, despite being mainly applied to ECC. However, the statistical distributions of matrix strength, fiber distribution, etc., must be proved based on experimental results. In addition, some mechanisms evidenced in experimental tests were not included in models, such as the fiber group effect; fiber rupture; actual distribution; and size of flaws to determine crack strength, scale effect, and fiber orientation and distribution. Furthermore, fiber orientation is typically ideally adopted with 3D and 2D patterns. Finally, some experimental studies were found testing SHCC under high temperatures. For such cases, no prediction models were found in the literature.

A fundamental topic that needs attention is practical recommendations on the residual capacity for typical crack width limits and ultimate strain under tension for the structural design of elements. In addition, the combination of SHCC with a low reinforcement ratio has recently shown low ductility (Shao and Billington (2022) [88]), which needs more experimental investigations.

The mentioned alternative methods for predicting SHCC behavior, such as multiphase modeling and machine learning approaches, are also robust material design and optimization techniques. However, they still require deeper consideration of the micro- and mesophenomena observed in experiments. Thus, combining more than one approach can be valuable for the further development of SHCC.

## 5. Final Remarks

The knowledge of the parameters governing the tensile response of SHCC is essential for boosting the production of such material with the desired performance to design resilient, sustainable, and durable structures, which are the main advantages of SHCCs. Engineers demand models to simulate structural elements or retrofit based on the evidenced experimental behavior. Thus, equations and numerical models are needed considering the experimental meso- and micromechanical finds. Furthermore, the design of strain hardening behavior with cementitious materials requires material optimization, based mainly on tensile behavior with goals to produce more sustainable and durable SHCCs. Hence, the present review aimed to bring the state of the art in modeling the tensile response of SHCC material, which has excellent potential for application in seismic and environmental aggressive regions, and highlight the experimental parameters that were proven to influence its behavior, such as group effect and fiber distribution. However, some are not considered in the models, revealing a gap for development in such research and practical fields. From the very robust techniques developed by research groups, it has been shown that parameters such as matrix strength variation are better represented considering spherical penny-shaped flaws within a normal distribution. Crack faces considered with nonzero stress are also more representative of reality due to the snubbing effect. It was further evidenced statistically that fiber strength and content are the most sensitive parameters in changing the strain capacity of the composite (Li, Weng and Yang (2019) [79]). More recently, a study proved that the influence of fiber flexural stiffness could better predict the ultimate strain of SHCC (Yao and Leung (2020) [70]). It should be mentioned that the field is currently under development. Despite the robust models, more research proving the effectiveness of the models based on experimental tests is necessary to consolidate the field and bring the outstanding behavior of SHCCs to practicing engineers. Experimental research evidencing statistical patterns of the variables most influencing mechanical behavior in different scales is necessary. Another essential aspect deserving attention is the application of SHCCs in structural components and the interaction of the composite material with steel and noncorrosive reinforcements—for example, the behavior of SHCCs in reinforced concrete beams failing in flexure. The beams are improved under service load levels, but the ductility, fundamental for resilient design, can be reduced in the case of underreinforced beams.

## Figures and Tables

**Table 1:** Influence of the casting method on the orientation coefficient.

Author	Comments	*η_θ_*
Duque and Graybeal (2017) [4]	Sample extracted from the slab perpendicular to flow direction	0.65
	Sample extracted from the slab at 45° to flow direction	0.74
	Sample extracted from the slab parallel to the flow direction	0.83
	Conventional molding	0.85
Kang and Kim (2011) [50]	The cast position was parallel to the tensile stress direction	0.645
	The cast position was transversal to the tensile stress direction	0.431
Abrishambaf, Pimentel, and Nunes (2019) [17]	Well-oriented (mold parallel to the induced electromagnetic field—1.5% of fiber content)	0.89
	Not oriented (mold orthogonal to the induced electromagnetic field—1.5% of fiber content)	0.71
	Well-oriented (3% of fiber content)	0.87
	Not oriented (3% of fiber content)	0.74

**Table 2:** Results of $\int_{0}^{\pi /2}p(\theta )g(\theta )\cos(\theta )d\theta$.

*η_θ_*	*θ*_mean_	$\int_{0}^{\pi /2}p(\theta )g(\theta )\cos(\theta )d\theta$
1	0°	1.00
0.966	15°	1.52
0.866	30°	1.73
0.707	45°	1.38
0.500	60°	0.94
0.259	75°	0.48
0	90°	0

## Data Availability

Not applicable.
